# Association between problematic smartphone use and gaming and disruptive behavioral disorder symptoms among Korean adolescents: a nationwide representative study

**DOI:** 10.3389/fpsyt.2026.1843287

**Published:** 2026-06-24

**Authors:** Yoo Jeong Lee, In Cheol Hwang, Hong Yup Ahn

**Affiliations:** 1 Department of Family Medicine, Korea University Guro Hospital, Seoul, Republic of Korea; 2 Department of Family Medicine, Gil Medical Center, Gachon University College of Medicine, Incheon, Republic of Korea; 3 Department of Statistics, Dongguk University, Seoul, Republic of Korea

**Keywords:** ADHD, conduct disorder, disruptive behavioral disorder, Korean adolescents, parental factors, problematic gaming, problematic smartphone use, urban-rural differences

## Abstract

**Background:**

Problematic smart device use is a growing public health concern among adolescents and is often associated with disruptive behavioral disorders (DBDs). However, little is known about how these associations depend on parental and residential factors in nationally representative populations.

**Methods:**

Data were obtained from the 2022 Korean National Mental Health Survey of Children and Adolescents. The analysis was performed on the data of adolescents aged 12–17 years who completed the DBD questionnaire (n=3,084 for problematic smartphone use [PSU]; n=2,393 for problematic gaming [PG]). Multivariable logistic regression models were used to examine overall associations and associations in residency and parental characteristic subgroups.

**Results:**

Adolescents with PSU or PG showed significantly higher prevalence of all DBD subtypes (all p<0.01). Distinct subgroup patterns emerged: PSU was associated with conduct disorder (CD) only in urban areas (odds ratio [OR]=2.03, 95% confidence interval [CI]=1.30–3.16), whereas PG was more strongly associated with ADHD-inattentive symptoms in rural areas (rural: OR = 4.22, 95% CI = 2.15–8.30; urban: OR = 2.10, 95% CI = 1.49–2.96). The PSU-oppositional defiant disorder association was stronger among adolescents with highly educated mothers (OR = 3.64 vs. 1.93), while the PSU-CD link was evident only when fathers drank less frequently (OR = 2.15, 95% CI = 1.34–3.45).

**Conclusions:**

PSU and PG show robust associations with all DBD subtypes, with distinct urban-rural and parental moderation patterns. If causal relationships are confirmed, targeted, family-based, and region-specific interventions may promote adolescent behavioral health at the national level.

## Introduction

Adolescent problematic smartphone use (PSU) and problematic gaming (PG) have emerged as critical global issues, and profoundly impact mental health and behavioral development (1, 2). Disruptive behavioral disorders (DBDs), which include oppositional defiant disorder (ODD), conduct disorder (CD), and attention-deficit/hyperactivity disorder (ADHD), are increasingly recognized as issues of significant public health concern, and digital overuse has been identified as a key modifiable correlate (3).

PSU refers to excessive, poorly controlled, and compulsive smartphone use that interferes with daily functioning, interpersonal relationships, academic performance, or psychological well-being (4). PG describes a pattern of persistent or recurrent gaming behavior characterized by impaired control, prioritization of gaming over other activities, and continuation despite negative consequences—broadly consistent with the ICD-11 criteria for gaming disorder, though the present study employs the broader term ‘problematic gaming’ to encompass sub-threshold cases not meeting full diagnostic criteria (5). The adolescent period represents a particularly vulnerable developmental window: neural circuits governing impulse control, reward processing, and social cognition are still maturing, rendering adolescents especially susceptible to the habit-forming properties of digital technologies. Consequently, PSU and PG during adolescence have been linked to a range of adverse outcomes, including academic underachievement, sleep disturbances, social withdrawal, and externalizing behavioral problems.

In Korea, national surveys report that over 20% of adolescents exhibit PSU, and PG rates approach 10% (6). These behaviors often co-occur with externalizing symptoms such as defiance and aggression, which reflect global trends (7). Furthermore, excessive screen engagement is hypothesized to be associated with altered prefrontal reward circuits and possibly related to impulsivity and antisocial behaviors (8), though it should be added that the directionality of these associations remains unclear.

Theoretically, PSU/PG-DBD associations may arise through multiple mechanisms. The Interaction of Person-Affect-Cognition-Execution (I-PACE) model (9) posits that problematic internet use emerges due to interactions among predisposing factors (e.g., ADHD, impulsivity), affective/cognitive responses to digital cues (craving, attentional bias), and executive deficits in inhibitory control. From a family systems perspective (10), parental factors may function as moderators—rather than mere confounders—of PSU/PG-DBD associations. A confounder is a common cause of both exposure and outcome and, if unadjusted, produces spurious associations; a moderator, by contrast, specifies the conditions under which an exposure–outcome relationship is stronger or weaker, and is of substantive theoretical interest (11). We treat parental education, drinking, and smoking as hypothesized moderators because: (1) these variables were selected *a priori* based on family systems theory, not *post-hoc*; (2) we formally tested exposure × moderator interaction terms in all models; and (3) the theoretical mechanism posits that parental characteristics change the strength of the digital overuse–behavioral dysregulation pathway (e.g., higher maternal education may amplify detection of PSU-related behavioral problems, while paternal alcohol use may disrupt the protective family environment that otherwise buffers against externalizing behaviors), rather than simply confounding it. Nonetheless, we acknowledge that these variables are also adjusted as covariates in the main-effects models to address their potential confounding role. Environmental affordances also matter; urban settings provide greater access to deviant peer networks and cyberbullying platforms, whereas rural isolation may amplify gaming’s appeal as escapism, given limited prosocial alternatives. These multi-level mechanisms suggest that PSU/PG-DBD links likely reflect transactional processes rather than simple unidirectional causation.

Longitudinal studies have recently adumbrated the temporal dynamics between digital overuse and behavioral problems. Boer et al. demonstrated bidirectional associations between ADHD symptoms and social media use over adolescence, and reported stronger effects from ADHD to social media than the reverse (12). Richard et al. systematically reviewed longitudinal evidence on gaming and conduct problems, and concluded pre-existing externalizing behaviors often precede gaming problems, suggesting a selection effect (13). Similarly, Ko et al. showed that ADHD symptoms predict later gaming disorder via impaired self-control and aggression, which highlighted potential mediating pathways (14). Poulain et al. (15) confirmed bidirectional smartphone-behavior difficulty associations over twelve months in German adolescents. These longitudinal findings underscore the complexity of PSU/PG–DBD relationships and the need for cross-sectional studies, like the present study, to be interpreted cautiously regarding causality. However, given the cross-sectional nature of the present study, all associations reported herein should be interpreted as correlational; bidirectional or reverse-causal pathways cannot be excluded.

Despite established links between PSU/PG and DBDs (16, 17), few nationally representative studies have examined how these associations are influenced by parental factors (e.g., maternal education, paternal drinking) and residency—critical modifiers within family systems theory. Utilizing the 2022 Korean National Mental Health Survey data, this study examines adjusted associations between PSU/PG and symptomatic DBDs with the aim of elucidating urban-rural and parent-specific patterns to provide a basis for targeted prevention strategies. Specifically, this study addresses three research questions: (1) Are PSU and PG independently associated with DBD subtypes after comprehensive covariate adjustment? (2) Do these associations vary by residency (urban vs. rural)? (3) Do parental factors (education, drinking, smoking) moderate PSU/PG-DBD relationships?

## Methods

### Design and participants

Data were drawn from the 2022 National Mental Health Survey of Children and Adolescents in Korea, a cross-sectional, nationally representative study employing stratified cluster sampling (6). The survey was conducted from September 2022 to February 2023 and included 6,275 participants aged 6–17 years (response rates: 59.94%). The DBD questionnaire was administered only to adolescents. After excluding 298 participants who had a current psychiatric diagnosis (specifically, depressive disorders, bipolar and related disorders, anxiety disorders, schizophrenia spectrum and other psychotic disorders, disruptive, impulse-control, and conduct disorders, substance-related and addictive disorders, feeding and eating disorders, elimination disorders, trauma- and stressor-related disorders, and suicide-related behaviors as documented in the survey’s clinical assessment module), the data from 3,084 adolescents (aged 12–17 years) were analyzed. This exclusion criterion was applied to reduce confounding by severe comorbid psychopathology that may independently elevate DBD symptom ratings. There were no missing data for DBD outcomes or PSU; however, 691 participants had missing data for PG and were excluded from PG analyses only, resulting in a final analytic sample of n=3,084 for PSU and n=2,393 for PG. The smaller sample size for PG analyses reflects the fact that 691 participants (22.4%) did not complete the gaming questionnaire. Comparative analysis showed that this group had a significantly higher proportion of girls (82.9% vs. 42.5% in the PG analysis sample, p<0.001), consistent with well-documented gender differences in gaming participation. Other baseline differences between groups (age, household income, father’s smoking, and some DBD symptoms) appeared to be secondary to this gender composition difference (**Supplementary Table 1**). All PG analyses were adjusted for sex and other relevant covariates to account for these differences. Trained interviewers administered the questionnaire using tablet PCs during household visits. Respondent privacy was ensured by keeping the tablet screen hidden from others.

### Measures

The primary outcomes were the symptomatic DBDs, assessed using the Korean version of the DBDs Rating Scale, DSM-5 version—Parent form (K-DBDRS). The K-DBDRS comprises 41 items rated on a 4-point Likert scale (0=not at all; 3=very much) that covers four subtypes: attention-deficit hyperactivity disorder inattention symptoms (ADHD-IA; 9 items), ADHD-hyperactivity/impulsivity symptoms (ADHD-HI; 9 items), oppositional defiant disorder (ODD; 16 items), and conduct disorder (CD; 7 items). Subtype scores were calculated as means, and participants were classified as symptomatic if they exceeded the cut-off values established in the validation study by Lee et al. (18): ADHD-IA ≥1.82, ADHD-HI ≥1.85, ODD ≥1.80, and CD ≥1.83 (mean item scores on the respective subscales).

The primary independent variables were PSU and PG. PSU was assessed using the short version of the Smartphone Addiction Scale (SAS-SV), a validated 10-item self-report measure. Items were rated on a 6-point Likert scale (1=strongly disagree; 6=strongly agree); total scores ranging from 10 to 60. Participants were classified as having PSU if their total scores exceeded the gender specific cut-offs (≥31 for boys; ≥33 for girls), determined by receiver operating characteristic curve analysis (4). The Structured Clinical Interview for Internet Gaming Disorder (SCI-IGD) was used to define PG (19). The SCI-IGD is a clinician-administered structured interview comprising 9 items corresponding to the nine DSM-5 criteria for Internet Gaming Disorder. Each item is rated dichotomously (present/absent) based on the past 12 months. Participants meeting 5 or more of the 9 criteria are classified as high-risk, yielding a binary indicator used in the present study. Due to copyright restrictions on the original developer’s materials, individual item wording is not publicly reproducible; however, the instrument’s criterion-based structure, inter-rater reliability, and convergent validity have been documented in the original validation study (19). It is important to note the term ‘PG’ used throughout this manuscript refers to a high-risk pattern of gaming behavior as operationalized by the SCI-IGD binary indicator, and does not imply a formal diagnosis of gaming disorder as defined by the ICD-11 or internet gaming disorder as described in the DSM-5. Covariates included participant age, sex, household income, residency, parental education level, and parental health-related habits (drinking and smoking).

### Statistical analysis

Results are presented as means ± standard deviations or frequencies (percentages). Inter-group differences were determined using t-tests or chi-square tests. Multivariable logistic regression, adjusted for covariates, was used to assess associations between PSU/PG and symptomatic DBDs in the overall and subgroups. For subgroup analyses, we tested interactions between each exposure (PSU, PG) and each moderator (sex, household economic status, residency, maternal education, paternal education, maternal drinking, paternal drinking, paternal smoking) for each outcome (ADHD-IA, ADHD-HI, ODD, CD), totaling 64 interaction terms. The analysis was conducted using Stata version 17.0 (StataCorp, College Station, TX, USA) using a significance level of α=0.05.

Subgroup analyses involving interaction terms were considered exploratory in nature. Given the correlated structure of the four DBD outcomes, a Bonferroni correction was deemed overly conservative; therefore, a conventional significance level of α=0.05 was maintained as the primary threshold. Of the 64 interaction terms tested, 4 (6.3%) reached conventional significance at α=0.05 (**Supplementary Table 2**, **S3**). The four significant interactions are theoretically coherent and directionally consistent with *a priori* hypotheses, lending additional credibility beyond chance alone.

Covariates were selected *a priori* based on theoretical considerations and prior empirical evidence. Participant age and sex were included as established demographic correlates of both DBD and digital overuse. Household income and parental education level were included as socioeconomic indicators known to influence both digital access and DBD risk. Residency (urban vs. rural) was included given its hypothesized role as an environmental moderator. Parental health-related habits (drinking and smoking) were included based on family systems theory, which posits that parental substance use disrupts family functioning and increases child vulnerability to behavioral dysregulation (10). All covariates were entered simultaneously in a single-step method. No stepwise or data-driven variable selection procedures were used, in order to minimize overfitting and maintain consistency with the *a priori* theoretical framework.

The present analyses did not incorporate survey weights or design-based standard errors, which represents a limitation. Given the stratified cluster sampling design, standard errors from unweighted logistic regression may be underestimated, potentially inflating statistical significance. Readers should interpret the reported confidence intervals and p-values with this caveat in mind.

## Results

In the overall analytic sample, the prevalence of PSU was 9.0% (n=277 of 3,084) and the prevalence of PG was 9.4% (n=224 of 2,393). The general characteristics of participants according to problematic smartphone use and gaming are summarized in **Table 1**. Problematic smartphone users were more likely to live in rural areas than non-problematic users (26.0% vs. 18.3%, *p* = 0.002), whereas PG was markedly more prevalent among boys. In the PSU and PG groups, adolescents with problematic use tended to have parents with lower educational levels (high school graduate or lower) (mothers: 50.6% vs. 39.8%, *p* = 0.001; fathers: 36.3% vs. 28.3%, *p* = 0.007 for smartphone use; mothers: 49.5% vs. 40.2%, *p* = 0.008; fathers: 41.2% vs. 28.1%, *p* < 0.001 for gaming), and parents who more frequently drink and smoke (all *p* < 0.01). Furthermore, problematic smartphone users and problematic gamers showed significantly higher rates of all four subtypes of symptomatic DBD than their non-problematic counterparts (all *p* < 0.01).

**Table 1:** General characteristics of the participants according to problematic behaviors.

Variables	Smartphone use			Gaming		
	Non-problematic	Problematic	*P*-value	Non-problematic	Problematic	*P*-value
Number	2,807	277		2,169	224	
Age, years	14.8 ± 1.6	14.8 ± 1.6	0.355	14.7 ± 1.6	14.7 ± 1.6	0.825
Sex						
Boy	1,349 (48.1)	144 (52.0)	0.212	1,186 (54.7)	189 (84.4)	<0.001
Girl	1,458 (51.9)	133 (48.0)		983 (45.3)	35 (15.6)	
Household income, Korean Won						
≥5 million	1,786 (63.6)	169 (61.0)	0.389	1,357 (62.6)	126 (56.3)	0.064
<5 million	1,021 (36.4)	108 (39.0)		812 (37.4)	98 (43.8)	
Residence						
Urban	2,293 (81.7)	205 (74.0)	0.002	1,769 (81.6)	175 (78.1)	0.210
Rural	514 (18.3)	72 (26.0)		400 (18.4)	49 (21.9)	
Parent education level						
≤ high school graduate of mother	1,095 (39.8)	136 (50.6)	0.001	852 (40.2)	108 (49.5)	0.008
≤ high school graduate of father	743 (28.3)	95 (36.3)	0.007	570 (28.1)	86 (41.2)	<0.001
Parent habits						
Mother’s drinking ≥ 2 times/month	702 (25.4)	103 (38.3)	<0.001	566 (26.6)	76 (34.9)	0.009
Father’s drinking ≥ 2 times/week	594 (22.5)	113 (43.1)	<0.001	473 (23.2)	77 (36.8)	<0.001
Father - current smoker	1,133 (42.9)	149 (56.9)	<0.001	900 (44.1)	121 (57.9)	<0.001
Symptomatic DBD						
ADHD-IA	647 (23.1)	122 (44.0)	<0.001	547 (25.2)	102 (45.5)	<0.001
ADHD-HI	541 (19.3)	100 (36.1)	<0.001	425 (19.6)	95 (42.4)	<0.001
ODD	673 (24.0)	127 (45.9)	<0.001	536 (24.7)	109 (48.7)	<0.001
CD	225 (8.0)	37 (13.4)	0.002	169 (7.8)	36 (16.1)	<0.001

DBD, disruptive behavior disorder; ADHD, attention-deficit/hyperactivity disorder; IA, inattention symptoms; HI, hyperactivity/impulsivity symptoms; ODD, oppositional defiant disorder; CD, conduct disorder. P-values were from t-test or chi-squared test.

**Figure 1:**
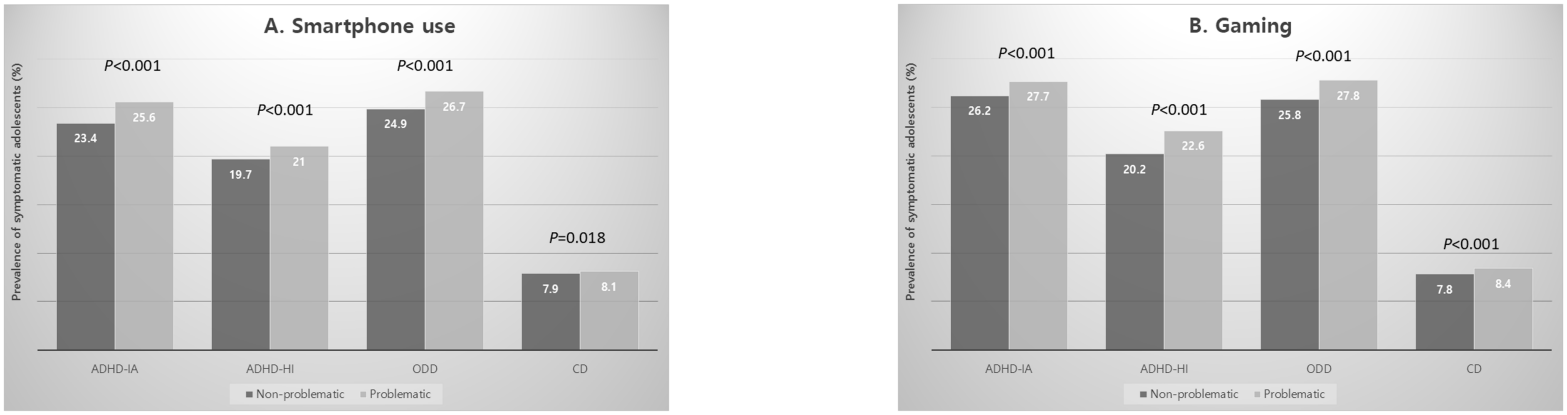
Adjusted prevalence of symptomatic disruptive behavior disorder subtypes by problematic device use. **(A)** Problematic smartphone use; **(B)** Problematic gaming. ADHD, attention-deficit/hyperactivity disorder; IA, inattention symptoms; HI, hyperactivity/impulsivity symptoms; ODD, oppositional defiant disorder; CD, conduct disorder. P-values were from multivariate logistic regression analysis adjusted for participant age and sex, household economic status, residency, parental education level, and parental health-related habits.

The fully adjusted prevalence of symptomatic DBD by PSU (A) and PG (B) are shown in **Figure 1**. All associations were significant, and showed that adolescents with PSU or PG had higher prevalences of all four DBD types than their non-problematic counterparts.

The differential associations between problematic device use and symptomatic DBDs across subgroups are provided in **Table 2**; only significant interaction effects are shown. Regarding area of residency, opposite patterns were observed for PSU and PG. The association between PSU and CD was significant only in urban areas (OR = 2.03, 95% CI = 1.30–3.16, *p* = 0.002), whereas the association between PG and ADHD-IA was stronger in rural than in urban areas (OR 4.22 vs. 2.10). In addition, the link between PSU and ODD was more pronounced among adolescents whose mothers had a higher education (OR 3.64 vs. 1.93), and the association between PSU and CD was evident only when fathers drank less frequently (OR = 2.15, 95% CI = 1.34–3.45, *p* = 0.002).

**Table 2:** Subgroup analyses of the relationship between problematic device use and symptomatic disruptive behavior disorders.

Subgroups	Smartphone–ODD		Smartphone–CD		Gaming–ADHD-IA	
	OR^a^ (95% CI)	P-value	OR^a^ (95% CI)	P-value	OR^a^ (95% CI)	P-value
Residency						
Urban			2.03 (1.30–3.16)	0.002	2.10 (1.49–2.96)	<0.001
Rural			0.61 (0.20–1.89)	0.395	4.22 (2.15–8.30)	<0.001
Mother’s education						
High	3.64 (2.50–5.31)	<0.001				
Low	1.93 (1.32–2.83)	0.001				
Father’s drinking						
< 2 times/week			2.15 (1.34–3.45)	0.002		
≥ 2 times/week			0.88 (0.40–1.96)	0.756		

OR, odds ratio; CI, confidence interval; ODD, oppositional defiant disorder; CD, conduct disorder; ADHD-IA, attention-deficit/hyperactivity disorder-inattention symptoms.

Only significant interaction effects (p<0.05) are presented. A total of 64 interaction terms were tested (2 exposures [PSU, PG] × 8 moderators [sex, economic status, residency, maternal education, paternal education, maternal drinking, paternal drinking, paternal smoking] × 4 outcomes [ADHD-IA, ADHD-HI, ODD, CD]); 4 (6.3%) reached conventional significance. Full results for all 64 interaction terms are provided in **Supplementary Table 1** and **S2**.

^a^ From multivariate logistic regression analysis adjusted for child’s age and sex, household economic status, residency, parental education level, parental health-related habits.

The differential associations between problematic device use and symptomatic DBDs across subgroups are provided in **Table 2**; only significant interaction effects are shown. Full regression results, including non-significant interactions, are presented in **Supplementary Table 2** and **S3**.

## Discussion

This nationally representative Korean study examined the associations between PSU/PG and symptomatic DBD in adolescents, and also investigated subgroup variations by sociodemographic and parental factors. The study revealed consistently higher DBD prevalence across all subtypes among problematic adolescent users and distinct patterns, such as PSU-CD links in urban areas and PG-ADHD-IA in rural settings, and identified modifiers like mothers’ education and fathers’ drinking. Given confirmation by future longitudinal studies of causal pathways from PSU and PG to the symptoms of DBD, our findings suggest several targeted intervention strategies. Specifically, for urban adolescents with PSU-CD patterns, school-based digital literacy curricula addressing cyberbullying, peer pressure, and healthy smartphone boundaries (20), and for rural adolescents with PG-ADHD patterns, cognitive-behavioral therapy and multi-level counseling interventions (21). Implementation considerations include cost-effectiveness, cultural adaptation to Korean contexts, and potential unintended consequences (e.g., stigma, digital divide exacerbation).

Crucially, the cross-sectional design of this study precludes any determination of causal direction. The observed associations between PSU/PG and DBD subtypes may reflect (1) digital overuse exacerbating behavioral difficulties, (2) pre-existing DBD symptoms driving increased digital engagement, or (3) shared underlying factors (e.g., temperamental impulsivity, family dysfunction) simultaneously elevating both risks. Recent longitudinal evidence suggests bidirectional relationships: while problematic digital use may exacerbate behavioral difficulties through reward dysregulation and attentional hijacking (15). In addition, recent studies also demonstrate that pre-existing conduct problems often precede gaming problems (13), and ADHD symptoms predict later gaming disorders via impaired self-control (14). Additionally, common underlying factors, such as temperamental impulsivity, family dysfunction, or peer deviance, may simultaneously drive digital overuse and DBD symptoms (15). Future longitudinal research with multiple assessment waves is essential to disentangle these complex temporal relationships and identify optimal intervention targets.

This study demonstrates robust associations between PSU/PG and all subtypes of symptomatic DBDs among Korean adolescents, even after adjusting for multiple sociodemographic and parental covariates. These findings align with recent longitudinal evidence linking smartphone addiction to externalizing behavioral difficulties via reward dysregulation and prefrontal impairment (15). The consistency across DBD subtypes and device types highlights the critical need for public health strategies that address problematic digital behaviors as potentially modifiable correlates. Implementing such strategies at a national level is essential for enhancing the scalability of interventions for adolescent psychopathology.

Our findings can be interpreted within the I-PACE model framework (9). First, regarding predisposing factors, the associations between PSU/PG and all DBD subtypes suggest that adolescents with pre-existing impulsivity and executive deficits—core features of ADHD and ODD—may be particularly vulnerable to developing problematic digital behaviors. Second, the stronger PG–ADHD-IA association in rural areas may reflect heightened affective/cognitive responses to gaming cues: rural adolescents with limited prosocial alternatives may develop stronger attentional biases toward gaming stimuli as a primary source of reward and escapism. Third, the urban PSU–CD pattern may involve executive deficits in inhibitory control, as urban environments provide more opportunities for smartphone-mediated antisocial interactions (e.g., cyberbullying, deviant peer contact) that require intact prefrontal regulation to resist. However, the cross-sectional design prevents us from determining whether DBD symptoms represent predisposing factors that increase PSU/PG risk, consequences of prolonged digital overuse, or both in a reciprocal cycle consistent with the I-PACE model’s process character.

Intriguing urban-rural disparities emerged in the subgroup analyses. PSU was significantly associated with CD among urban adolescents, whereas PG exhibited a markedly stronger relationship with ADHD-IA in rural areas. We tentatively propose that the urban-specific PSU–CD association may reflect contextual factors unique to urban environments, such as greater exposure to deviant peer networks and digital platforms (22). However, we emphasize that these are *post-hoc* hypotheses not directly testable with the present data, and alternative explanations—including reverse causation (CD symptoms driving PSU) or unmeasured urban-specific confounders—cannot be excluded. Longitudinal studies with mediation analyses incorporating peer network measures and urban-specific contextual variables are needed to evaluate these proposed pathways in Korean adolescents. However, alternative explanations cannot be ruled out, including the possibility that CD symptoms drive PSU or that common underlying factors, such as family dysfunction, contribute to both PSU and PG.

Conversely, rural adolescents with limited extracurricular alternatives and fewer social outlets may turn to gaming as a primary form of escapism. This may render these adolescents particularly vulnerable to attention deficits, as prolonged, solitary gaming sessions have been linked to disrupted cognitive control and reward processing (23), potentially via altered frontostriatal network function (24). The OR of 4.22 for the association between PG and ADHD-IA in rural areas is therefore particularly striking and warrants careful clinical contextualization. However, this large effect size should be interpreted with caution given the small subgroup sample size: among the rural subsample (n=449), 49 adolescents were classified as PG-positive, of whom 27 adolescents met criteria for symptomatic ADHD-IA. The wide confidence interval for this estimate reflects limited precision, and the finding should be considered hypothesis-generating rather than definitive. Replication in larger rural samples is needed before this association can inform clinical practice. Rural adolescents in Korea face a compounded vulnerability: they are more likely to rely on gaming as a primary recreational outlet due to limited alternatives, while simultaneously having substantially reduced access to diagnostic and mental health services, which remain disproportionately concentrated in urban centers. This structural inequity means that even when PG-ADHD associations are recognized, the pathway from problem recognition to appropriate treatment is considerably longer for rural youth. Our findings thus underscore an urgent need for geographically tailored responses, including telemedicine-based screening, mobile mental health outreach, and community-level interventions specifically targeting rural Korean adolescents with high-risk gaming behaviors (21, 22). Taken together, these residence-specific patterns highlight how environmental affordances shape digital overuse pathways: urban settings may amplify externalizing behaviors via smartphone-mediated social interaction, whereas rural isolation may intensify the attentional vulnerabilities associated with problematic gaming.

The stronger association between PSU and ODD in adolescents with highly educated mothers warrants careful interpretive caution. Two distinct explanations must be distinguished. First, this pattern may reflect differential reporting rather than true effect modification: mothers with higher educational attainment may be more attuned to detecting and reporting subtle oppositional behaviors in their children, inflating the observed PSU–ODD association in this subgroup through a reporting bias mechanism (25). If this is the case, the finding has implications for measurement validity rather than intervention targeting—specifically, it suggests that ODD may be underdetected in less-educated families, and that standardized behavioral screening tools should be employed regardless of parental education level. Second, a true moderation effect remains possible: higher maternal education may be associated with greater parental monitoring of smartphone use, which paradoxically amplifies adolescent resistance and oppositional behavior when limits are imposed (25). Disentangling these two explanations requires multi-informant assessment designs (e.g., teacher and clinician ratings in addition to parent reports) and is an important direction for future research. Conversely, the significant link between PSU and CD observed only in groups with low paternal alcohol consumption indicates that stable paternal figures in less alcohol-disrupted households may serve as protective factors against externalizing disorders (26). These parent-specific differences underscore the need for family-centered interventions targeting maternal education and paternal drinking patterns to mitigate gaming addiction risks in adolescents.

Our findings must be interpreted in the Korean cultural context, where intense academic pressure, widespread PC bang (gaming café) culture, and high smartphone penetration create unique risk environments (27). Government policies such as the “Shutdown Law” (a midnight to 6 am gaming ban for minors, later repealed) and current internet/smartphone addiction prevention initiatives reflect national concern, yet evidence for their effectiveness remains limited (27). Equity and stigma considerations are paramount: labeling adolescents as having “problematic” or “addictive” behaviors risks stigmatization and may deter help-seeking (28), and our finding that PSU and PG are more common among adolescents with less-educated parents raises concerns that interventions requiring parental resources (time, digital literacy, financial capacity) may widen disparities unless designed with universal access in mind. Unintended consequences of restrictive interventions also warrant attention, as excessive parental control over digital devices may damage parent-child relationships, drive secretive use, or deprive adolescents of beneficial online socialization, which is particularly important for rural youth with limited offline peer access (29). Balanced approaches that promote healthy digital habits rather than abstinence and involve adolescents in co-designing solutions, may be more acceptable and sustainable. International comparisons reveal both universal and culture-specific patterns: while PSU and PG-externalizing links are documented globally (7, 17), the specific moderating roles of maternal education and paternal drinking may reflect Korean gender norms around parenting responsibilities and drinking culture; cross-national replication in diverse contexts is needed.

This study has several limitations. First, its cross-sectional design precludes causal inference regarding the directionality between PSU/PG and DBD symptoms; prospective studies are needed to clarify bidirectional or mediating pathways. Second, reliance on parent-reported DBD ratings and adolescent self-reports regarding PSU and PG introduces potential reporter biases. Specifically, the use of different informants for the exposure (adolescent self-report) and outcome (parent-report) measures may introduce systematic discrepancies. In particular, mothers with higher educational attainment may have greater sensitivity to detecting subtle behavioral symptoms in their children, which may have inflated the subgroup association between PSU and ODD in the higher maternal education group. Future studies should employ multi-informant assessment strategies, including teacher ratings and clinician-administered instruments, to minimize this source of bias. Third, 22.4% of participants did not complete the gaming questionnaire, resulting in a smaller sample for PG analyses. This missing data was not random but was strongly associated with female sex (82.9% of the PG-missing group were girls vs. 42.5% in the PG analysis sample), reflecting well-documented gender differences in gaming participation. While we adjusted for sex and other covariates in all PG analyses, residual confounding or selection bias cannot be entirely ruled out. The observed differences in other baseline characteristics (age, household income, father’s smoking, and some DBD symptoms) between groups appear to be secondary consequences of this gender imbalance rather than independent sources of bias. Nevertheless, our PG findings should be interpreted with this limitation in mind. Fourth, using a SCI-IGD–derived binary high-risk indicator to classify PG, imposed by item-level restrictions, may reduce granularity relative to continuous scoring methods. Finally, unmeasured confounders, such as screen time duration, co-occurring psychiatric conditions, or peer influences, could influence observed device-DBD links and warrant comprehensive adjustment in future research. Despite these constraints, the nationally representative sampling and covariate adjustments strengthen the generalizability of our findings in the Korean adolescent population.

## Data Availability

The original contributions presented in the study are included in the article/**Supplementary Material**, further inquiries can be directed to the corresponding author/s.

## References

[1] Cheng X Fan Y Li S Li X Jin S Zhou C . Research landscape and trends of internet addiction disorder: a comprehensive bibliometric analysis of publications in the past 20 years. Digit Health. (2025) 11:20552076251336940. doi: 10.1177/20552076251336940 40297375 PMC12034966

[2] Satapathy P Khatib MN Balaraman AK R R Kaur M Srivastava S . Burden of gaming disorder among adolescents: a systemic review and meta-analysis. Public Health Pract. (2025) 9:100565. doi: 10.1016/j.puhip.2024.100565 40115446 PMC11925544

[3] Thorell LB Burén J Ström Wiman J Bergman Nutley S de la Peña AG Servera M . Longitudinal associations between digital media use and ADHD symptoms in children and adolescents: a systematic literature review. Eur Child Adolesc Psychiatry. (2024) 33:2503–26. doi: 10.1007/s00787-022-02130-3 36562860 PMC11272698

[4] Kwon M Kim DJ Cho H Yang S . The smartphone addiction scale: development and validation of a short version for adolescents. PloS One. (2013) 8:e83558. doi: 10.1371/journal.pone.0083558 24391787 PMC3877074

[5] Yen JY Higuchi S Lin PY Lin PC Chou WP Ko CH . Functional impairment, insight, and comparison between criteria for gaming disorder in the International Classification of Diseases, 11 Edition and internet gaming disorder in Diagnostic and Statistical Manual of Mental Disorders, Fifth Edition. J Behav Addict. (2022) 11:1012–23. doi: 10.1556/2006.2022.00079 36326855 PMC9881664

[6] Kim BN Do R Sim H Kim S Park MH Kim JI . Epidemiological study of DSM-5 mental disorders: National mental health survey of Korea - child and adolescent 2022. Asian J Psychiatr. (2025) 107:104425. doi: 10.1016/j.ajp.2025.104425 40158275

[7] Sohn SY Rees P Wildridge B Kalk NJ Carter B . Prevalence of problematic smartphone usage and associated mental health outcomes amongst children and young people: a systematic review, meta-analysis and GRADE of the evidence. BMC Psychiatry. (2019) 19:356. doi: 10.1186/s12888-019-2350-x 31779637 PMC6883663

[8] Chen YY Yim H Lee TH . Negative impact of daily screen use on inhibitory control network in preadolescence: a two-year follow-up study. Dev Cognit Neurosci. (2023) 60:101218. doi: 10.1016/j.dcn.2023.101218 36821878 PMC9933860

[9] Brand M Wegmann E Stark R Müller A Wölfling K Robbins TW . The interaction of Person-Affect-Cognition-Execution (I-PACE) model for addictive behaviors: update, generalization to addictive behaviors beyond internet-use disorders, and specification of the process character of addictive behaviors. Neurosci Biobehav Rev. (2019) 104:1–10. doi: 10.1016/j.neubiorev.2019.06.032 31247240

[10] Nielsen P Favez N Rigter H . Parental and family factors associated with problematic gaming and problematic internet use in adolescents: a systematic literature review. Curr Addict Rep. (2020) 7:365–86. doi: 10.1007/s40429-020-00320-0 30311153

[11] Emsley R Dunn G White IR . Mediation and moderation of treatment effects in randomised controlled trials of complex interventions. Stat Methods Med Res. (2010) 19:237–70. doi: 10.1177/0962280209105014 19608601

[12] Boer M Stevens G Finkenauer C de Looze ME van den Eijnden RJ . Attention deficit hyperactivity disorder-symptoms, social media use intensity, and social media use problems in adolescents: investigating directionality. Child Dev. (2020) 91:e853–65. doi: 10.1111/cdev.13334 31654398 PMC7497191

[13] Richard J Temcheff CE Derevensky JL . Gaming disorder across the lifespan: a scoping review of longitudinal studies. Curr Addict Rep. (2020) 7:561–87. doi: 10.1007/s40429-020-00339-3 30311153

[14] Ko CH Lin HC Lin PC Yen JY . Validity, functional impairment and complications related to Internet gaming disorder in the DSM-5 and gaming disorder in the ICD-11. Aust Nz J Psychiat. (2020) 54:707–18. doi: 10.1177/0004867419881499 31631668

[15] Poulain T Vogel M Kliesener T Erbsmehl L Hilbert A Kiess W . Associations between changes in behavioral difficulties and levels of problematic smartphone use in adolescents over a 1-year period. Eur Child Adolesc Psychiatry. (2023) 32:533–6. doi: 10.1007/s00787-021-01874-8 34546407 PMC10038943

[16] Choi JY Kang JH . Longitudinal relationship between smartphone dependence and externalizing behavior problems: an autoregressive cross-lagged model. Psychiatry Investig. (2025) 22:287–92. doi: 10.30773/pi.2024.0375 40143725 PMC11962521

[17] Gonzalez-Bueso V Santamaría JJ Fernández D Merino L Montero E Ribas J . Association between Internet Gaming Disorder or pathological video-game use and comorbid psychopathology: a comprehensive review. Int J Environ Res Public Health. (2018) 15:0668. doi: 10.3390/ijerph15040668 29614059 PMC5923710

[18] Lee ES Ryu V Choi J Kim B Ha JH Hong M . Reliability and validity of the Korean version of Disruptive Behavior Disorders Rating Scale, DSM-5 Version-Parent Form. Psychiatry Investig. (2022) 19:884–97. doi: 10.30773/pi.2022.0112 36444152 PMC9708864

[19] Ko CH Yen JY Chen SH Wang PW Chen CS Yen CF . Evaluation of the diagnostic criteria of Internet gaming disorder in the DSM-5 among young adults in Taiwan. J Psychiatr Res. (2014) 53:103–10. doi: 10.1016/j.jpsychires.2014.02.008 24581573

[20] King DL Delfabbro PH Perales JC Deleuze J Király O Kuss DJ . Maladaptive player-game relationships in problematic gaming and gaming disorder: a systematic review. Clin Psychol Rev. (2019) 73:101777. doi: 10.1016/j.cpr.2019.101777 31707185

[21] Chang CH Chang YC Yang LK Chen YC Chien WC Lin CW . The comparative efficacy of treatments for children and young adults with internet addiction/internet gaming disorder: an updated meta-analysis. Int J Env Res Pub He. (2022) 19:2612. doi: 10.3390/ijerph19052612 35270305 PMC8909504

[22] Liu Y Ge X Wang Y Wang J Liang Y Xiao J . Urban-rural differences in the association between internet use trajectories and depressive symptoms in Chinese adolescents: longitudinal observational study. J Med Internet Res. (2025) 27:e63799. doi: 10.2196/63799 39919294 PMC11845883

[23] Li Q Wang Y Yang Z Dai W Zheng Y Sun Y . Dysfunctional cognitive control and reward processing in adolescents with internet gaming disorder. Psychophysiology. (2020) 57:e13469. doi: 10.1111/psyp.13469 31456249

[24] Schettler L Thomasius R Paschke K . Neural correlates of problematic gaming in adolescents: a systematic review of structural and functional magnetic resonance imaging studies. Addict Biol. (2022) 27:e13093. doi: 10.1111/adb.13093 34496459

[25] Liu MC Chang JC Lee CS . Interactive association of maternal education and peer relationship with oppositional defiant disorder: an observational study. BMC Psychiatry. (2021) 21:160. doi: 10.1186/s12888-021-03157-7 33752611 PMC7983394

[26] Long EC Lönn SL Sundquist J Sundquist K Kendler KS . The role of parent and offspring sex on risk for externalizing psychopathology in offspring with parental alcohol use disorder: a national Swedish study. Soc Psychiatry Psychiatr Epidemiol. (2018) 53:1381–9. doi: 10.1007/s00127-018-1563-5 30019183 PMC6252126

[27] Choi J Cho H Lee S Kim DJ Choi JY Choi SW . Effect of the online game shutdown policy on internet use, internet addiction, and sleeping hours in Korean adolescents. J Adolesc Health. (2018) 62:548–55. doi: 10.1016/j.jadohealth.2017.11.291 29434003

[28] Billieux J King DL Higuchi S Achab S Bowden-Jones H Clark VG . Functional impairment matters in the screening and diagnosis of gaming disorder. J Behav Addict. (2017) 6:285–9. doi: 10.1556/2006.6.2017.036 28816514 PMC5700712

[29] George MJ Russell MA Piontak JR Odgers CL . Concurrent and subsequent associations between daily digital technology use and high-risk adolescents' mental health symptoms. Child Dev. (2018) 89:78–88. doi: 10.1111/cdev.12819 28466466 PMC5670031

