# Supplementary material for: Association between problematic smartphone use and gaming and disruptive behavioral disorder symptoms among Korean adolescents: a nationwide representative study
Source: Front Psychiatry. 2026 Jun 24;17:1843287. doi: 10.3389/fpsyt.2026.1843287 (PMC13343219; doi:10.3389/fpsyt.2026.1843287)
Supplement: Supplementary file 1 [file Table1.docx]

**Supplementary Table S1.** General characteristics according to participating on problematic gaming analyses.

|  |  | Analyses on | |  |
| --- | --- | --- | --- | --- |
|  |  | Both PSU and PG | PSU only | *P*-value |
| Number | | 2,393 | 691 |  |
| Age, years | | 14.7±1.6 | 15.0±1.6 | <0.001 |
| Girl | | 1,018 (42.5) | 573 (82.9) | <0.001 |
| Household income of <5 million KRW | | 910 (38.0) | 219 (31.7) | 0.002 |
| Rural residence | | 449 (18.8) | 137 (19.8) | 0.530 |
| ≤ High school graduate of mother | | 960 (41.1) | 271 (39.7) | 0.536 |
| ≤ High school graduate of father | | 656 (29.3) | 182 (27.8) | 0.454 |
| Mother's drinking ≥ 2 times/month | | 642 (27.3) | 163 (23.8) | 0.068 |
| Father's drinking ≥ 2 times/week | | 550 (24.5) | 157 (23.9) | 0.769 |
| Father - current smoker | | 1,021 (45.4) | 261 (39.7) | 0.010 |
| *Symptomatic DBD* | |  |  |  |
|  | ADHD-IA | 649 (27.1) | 120 (17.4) | <0.001 |
|  | ADHD-HI | 520 (21.7) | 121 (17.5) | 0.016 |
|  | ODD | 645 (27.0) | 155 (22.4) | 0.017 |
|  | CD | 205 (8.6) | 57 (8.3) | 0.792 |

KRW, Korean Won; DBD, disruptive behavior disorder; ADHD, attention-deficit/hyperactivity disorder; IA, inattention symptoms; HI, hyperactivity/impulsivity symptoms; ODD, oppositional defiant disorder; CD, conduct disorder. PSU, problematic smartphone use; PG, problematic gaming. P-values were from t-test or chi-squared test.

**Supplementary Table S2.** Full logistic regression results for stratum-specific results and interaction terms between problematic smartphone use and moderators on symptomatic disruptive behavior disorder subtypes

|  |  | Odds ratio^a^ (95% confidence interval) | | | |
| --- | --- | --- | --- | --- | --- |
| Moderator | Level | ADHD-IA | ADHD-HI | ODD | CD |
| Sex | Boys | 2.08 (1.42–3.03)*** | 2.72 (1.85–4.01)*** | 2.08 (1.42–3.04)*** | 1.64 (0.92–2.94) |
|  | Girls | 3.13 (2.13–4.60)*** | 2.08 (1.38–3.13)*** | 3.48 (2.37–5.11)*** | 1.64 (0.92–2.91) |
|  | Interaction | 1.45 (0.85–2.46) | 0.70 (0.41–1.22) | 1.59 (0.94–2.71) | 1.02 (0.46–2.26) |
| Household income | ≥ 5 million KRW | 2.16 (1.55–3.03)*** | 2.18 (1.53–3.10)*** | 2.57 (1.84–3.58)*** | 1.89 (1.13–3.15)* |
|  | < 5 million KRW | 3.36 (2.12–5.32)*** | 2.79 (1.75–4.45)*** | 2.95 (1.86–4.67)*** | 1.26 (0.64–2.47) |
|  | Interaction | 1.49 (0.86–2.61) | 1.34 (0.76–2.36) | 1.11 (0.64–1.93) | 0.74 (0.32–1.69) |
| Residency | Urban | 2.39 (1.76–3.25)*** | 2.47 (1.80–3.39)*** | 2.66 (1.96–3.61)*** | 2.03 (1.30–3.16)** |
|  | Rural | 3.45 (1.94–6.15)*** | 2.28 (1.24–4.20)** | 2.88 (1.62–5.11)*** | 0.61 (0.20–1.89) |
|  | Interaction | 1.30 (0.70–2.45) | 0.92 (0.48–1.79) | 1.03 (0.55–1.95) | 0.31 (0.10–0.97)* |
| Mother’s education | ≥ college graduate | 2.74 (1.88–4.00)*** | 2.88 (1.95–4.26)*** | 3.64 (2.50–5.31)*** | 2.03 (1.13–3.66)* |
|  | ≤ high school graduate | 2.30 (1.57–3.38)*** | 1.94 (1.30–2.90)** | 1.93 (1.32–2.83)** | 1.31 (0.75–2.30) |
|  | Interaction | 0.89 (0.52–1.51) | 0.71 (0.41–1.22) | 0.54 (0.32–0.91)* | 0.66 (0.30–1.46) |
| Father’s education | ≥ college graduate | 2.87 (2.06–4.00)*** | 2.38 (1.69–3.37)*** | 2.92 (2.10–4.07)*** | 1.78 (1.07–2.97)* |
|  | ≤ high school graduate | 1.95 (1.23–3.10)** | 2.35 (1.46–3.80)*** | 2.25 (1.42–3.57)** | 1.44 (0.73–2.81) |
|  | Interaction | 0.72 (0.41–1.26) | 1.02 (0.57–1.82) | 0.74 (0.42–1.29) | 0.78 (0.34–1.79) |
| Mother’s drinking | < 2 times/month | 2.78 (1.99–3.89)*** | 2.31 (1.63–3.27)*** | 2.19 (1.57–3.07)*** | 1.59 (0.99–2.56) |
|  | ≥ 2 times/month | 2.11 (1.34–3.34)** | 2.47 (1.54–3.96)*** | 3.90 (2.47–6.17)*** | 1.94 (0.88–4.31) |
|  | Interaction | 0.78 (0.45–1.36) | 1.10 (0.62–1.96) | 1.67 (0.96–2.90) | 1.01 (0.41–2.49) |
| Father’s drinking | < 2 times/week | 2.90 (2.05–4.10)*** | 2.81 (1.97–4.01)*** | 2.95 (2.08–4.16)*** | 2.15 (1.34–3.45)** |
|  | ≥ 2 times/week | 1.99 (1.30–3.05)** | 1.87 (1.19–2.94)** | 2.30 (1.50–3.52)*** | 0.88 (0.40–1.96) |
|  | Interaction | 0.72 (0.41–1.24) | 0.66 (0.37–1.17) | 0.76 (0.44–1.30) | 0.39 (0.16–0.97)* |
| Father’s current smoking | No | 2.48 (1.65–3.72)*** | 2.53 (1.66–3.88)*** | 3.27 (2.20–4.88)*** | 1.60 (0.84–3.03) |
|  | Yes | 2.49 (1.73–3.57)*** | 2.32 (1.60–3.37)*** | 2.27 (1.58–3.27)*** | 1.66 (0.98–2.84) |
|  | Interaction | 1.09 (0.64–1.87) | 0.93 (0.53–1.62) | 0.68 (0.40–1.15) | 1.03 (0.45–2.34) |

KRW, Korean Won; ADHD, attention-deficit/hyperactivity disorder; IA, inattention symptoms; HI, hyperactivity/impulsivity symptoms; ODD, oppositional defiant disorder; CD, conduct disorder. ^a^From multivariate logistic regression analysis adjusted for child's age and sex, household economic status, residency, parental education level, parental health-related habits. *P<0.05; **P<0.01; ***P<0.001.

**Supplementary Table S3.** Full logistic regression results for stratum-specific results and interaction terms between problematic gaming and moderators on symptomatic disruptive behavior disorder subtypes

|  |  | Odds ratio^a^ (95% confidence interval) | | | |
| --- | --- | --- | --- | --- | --- |
| Moderator | Level | ADHD-IA | ADHD-HI | ODD | CD |
| Sex | Boys | 2.35 (1.68–3.29)*** | 2.98 (2.10–4.23)*** | 2.77 (1.98–3.89)*** | 2.40 (1.47–3.93)*** |
|  | Girls | 2.86 (1.40–5.86)** | 5.55 (2.67–11.54)*** | 3.43 (1.66–7.08)** | 3.00 (1.24–7.27)* |
|  | Interaction | 1.19 (0.54–2.60) | 1.76 (0.79–3.94) | 1.22 (0.55–2.68) | 1.22 (0.45–3.34) |
| Household income | ≥ 5 million KRW | 2.16 (1.47–3.19)*** | 3.06 (2.05–4.57)*** | 2.66 (1.80–3.92)*** | 1.73 (0.91–3.26) |
|  | < 5 million KRW | 2.82 (1.73–4.61)*** | 3.69 (2.23–6.13)*** | 3.24 (1.97–5.33)*** | 3.45 (1.87–6.39)*** |
|  | Interaction | 1.37 (0.74–2.52) | 1.26 (0.68–2.36) | 1.20 (0.65–2.20) | 2.21 (0.95–5.17) |
| Residency | Urban | 2.10 (1.49–2.96)*** | 3.13 (2.20–4.45)*** | 2.93 (2.08–4.13)*** | 1.82 (1.08–3.08)* |
|  | Rural | 4.22 (2.15–8.30)*** | 4.51 (2.27–8.97)*** | 2.55 (1.30–4.99)** | 5.45 (2.29–12.97)*** |
|  | Interaction | 2.11 (1.01–4.38)* | 1.45 (0.69–3.03) | 0.88 (0.42–1.82) | 2.36 (0.95–5.89) |
| Mother’s education | ≥ college graduate | 2.43 (1.59–3.73)*** | 3.60 (2.33–5.57)*** | 2.77 (1.80–4.25)*** | 1.53 (0.73–3.23) |
|  | ≤ high school graduate | 2.45 (1.59–3.79)*** | 3.19 (2.03–5.03)*** | 3.10 (2.00–4.81)*** | 3.19 (1.84–5.53)*** |
|  | Interaction | 1.06 (0.59–1.92) | 0.86 (0.47–1.57) | 0.99 (0.55–1.79) | 2.27 (0.93–5.56) |
| Father’s education | ≥ college graduate | 2.44 (1.65–3.60)*** | 3.31 (2.23–4.92)*** | 2.57 (1.74–3.80)*** | 1.63 (0.85–3.01) |
|  | ≤ high school graduate | 2.38 (1.46–3.87)** | 3.59 (2.14–6.03)*** | 3.48 (2.12–5.71)*** | 4.01 (2.13–7.54)*** |
|  | Interaction | 0.98 (0.53–1.80) | 0.99 (0.53–1.84) | 1.17 (0.64–2.15) | 2.32 (0.99–5.46) |
| Mother’s drinking | < 2 times/month | 2.70 (1.87–3.91)*** | 3.75 (2.57–5.48)*** | 2.99 (2.06–4.32)*** | 2.42 (1.47–3.96)*** |
|  | ≥ 2 times/month | 1.89 (1.10–3.25)* | 2.74 (1.58–4.77)*** | 2.65 (1.54–4.55)*** | 2.81 (1.15–6.86)* |
|  | Interaction | 0.74 (0.39–1.39) | 0.81 (0.43–1.55) | 0.91 (0.48–1.71) | 0.98 (0.38–2.57) |
| Father’s drinking | < 2 times/week | 2.79 (1.92–4.05)*** | 3.36 (2.29–4.95)*** | 3.28 (2.25–4.78)*** | 2.93 (1.79–4.78)*** |
|  | ≥ 2 times/week | 1.77 (1.05–3.00)* | 3.33 (1.94–5.71)*** | 2.16 (1.28–3.65)** | 1.59 (0.64–3.96) |
|  | Interaction | 0.67 (0.36–1.26) | 1.07 (0.57–2.03) | 0.68 (0.36–1.26) | 0.44 (0.17–1.18) |
| Father’s current smoking | No | 2.06 (1.29–3.27)** | 2.77 (1.69–4.53)*** | 2.54 (1.60–4.03)*** | 2.78 (1.43–5.40)** |
|  | Yes | 2.63 (1.75–3.95)*** | 3.84 (2.54–5.81)*** | 3.09 (2.06–4.65)*** | 2.31 (1.31–4.07)** |
|  | Interaction | 1.25 (0.68–2.27) | 1.55 (0.83–2.88) | 1.21 (0.67–2.20) | 0.99 (0.43–2.29) |

KRW, Korean Won; ADHD, attention-deficit/hyperactivity disorder; IA, inattention symptoms; HI, hyperactivity/impulsivity symptoms; ODD, oppositional defiant disorder; CD, conduct disorder. ^a^From multivariate logistic regression analysis adjusted for child's age and sex, household economic status, residency, parental education level, parental health-related habits. *P<0.05; **P<0.01; ***P<0.001.
